# Epidemiology and Outcome of Traumatic Brain Injuries: A Retrospective Study in a Tertiary Care Center

**DOI:** 10.7759/cureus.90510

**Published:** 2025-08-19

**Authors:** Rudra N Shah, Yam B Roka, Ashish J Thapa, Alok Jha, Chandan N Sah

**Affiliations:** 1 Neurosurgery, University Hospitals Coventry and Warwickshire NHS Trust, Coventry, GBR; 2 Neurosurgery, Gandaki Medical College, Pokhara, NPL; 3 Neurosurgery, Neuro Cardio and Multispeciality Hospital, Biratnagar, NPL; 4 Internal Medicine, Nepalgunj Medical College, Kohalpur, NPL

**Keywords:** epidemiology, glasgow coma scale, outcomes, public health, road traffic accidents, traumatic brain injury

## Abstract

Background: Traumatic brain injuries (TBI) are a major public health concern worldwide, particularly in low- and middle-income countries such as Nepal, where road traffic accidents (RTAs) are the leading cause. Despite advances in medical care, TBI remains a significant contributor to morbidity and mortality. This study aims to analyze the epidemiology, clinical presentation, and outcomes of TBI cases in a tertiary care center in Nepal.

Materials and methods: A retrospective study was conducted at Neuro Cardio & Multispeciality Hospital, Nepal, reviewing 1067 TBI patients admitted between 1^st^ August 2023 and 31^st^ July 2024. Patients were evaluated for demographic details and injury severity based on the Glasgow Coma Scale (GCS), radiological findings, hospital stay, and outcomes. Data were analyzed using SPSS (IBM Corp., Armonk, NY, USA), with multinomial logistic regression performed to assess the relationship between GCS on admission, hospital length of stay (LOS), and clinical outcomes (measured by the Glasgow Outcome Scale (GOS)).

Results: The majority of patients were male (73.9%) with RTAs accounting for 69.5% of TBIs and mild TBIs (GCS of 13-15) represented the majority (75.7%) of cases. Neurosurgical intervention was required in 18.1% of cases, with decompressive craniectomy being the most common procedure. The average ICU stay was eight days, and mortality was 7.2%. GCS on admission was a significant predictor of LOS and clinical outcomes (p<0.001).

Conclusion: The study highlights the importance of early intervention and standardized tools like GCS in predicting patient outcomes. Improved road safety measures and trauma care systems are essential for reducing the incidence and severity of TBIs in resource-limited settings.

## Introduction

Traumatic brain injury (TBI) is a significant global health issue that occurs when an external force leads to head trauma, resulting in varying degrees of neurological damage, with an estimated 69 million individuals affected annually worldwide [1]. TBIs contribute substantially to morbidity, disability, and mortality, particularly in low- and middle-income countries, where healthcare systems are often ill-equipped to manage severe cases. The World Health Organization (WHO) estimates that approximately 1.19 million people die each year as a result of road traffic accidents, with TBI being a major contributor to this burden [2,3]. In Nepal, where road infrastructure is often inadequate and enforcement of traffic laws is inconsistent, RTAs remain the predominant cause of TBIs [4].

The management of TBI presents complex challenges, particularly in resource-constrained environments. Specialized care, including neuroimaging, neurosurgery, and critical care, is often inaccessible to a large portion of the population in rural areas. The shortage of neurosurgeons and advanced trauma care facilities in many regions of Nepal exacerbates the problem, leading to higher rates of morbidity and mortality in TBI cases [5,6].

Despite advances in neurosurgical techniques and trauma care, the management of TBI continues to present challenges, particularly in resource-limited settings where access to specialized care is restricted [2]. The variation in outcomes among TBI patients across different regions highlights the importance of context-specific data on epidemiology and treatment [7]. Understanding the epidemiological patterns and the factors influencing outcomes is essential for shaping policy and improving care for TBI patients [8]. This study aims to analyze the epidemiology, clinical presentation, and outcomes of TBI cases admitted to a tertiary care center in Nepal, providing insights into the patterns and predictors of patient recovery. And, to assess the relationship between Glasgow coma scale (GCS) scores on admission and patient outcomes, including hospital length of stay (LOS) and Glasgow Outcome Scale (GOS) at discharge.

This article was previously presented as an oral presentation at the 11th National Conference of NESON Nepalese Society of Neurosurgery on 14th - 16th Nov, 2024.

## Materials and methods

This study was done in the Department of Neurosurgery at Neuro Cardio & Multispeciality Hospital, a tertiary care center in Nepal. This hospital provides emergency care to more than 0.5 million people annually in the eastern part of Nepal and bordering areas of India. This is a retrospective study of all the traumatic brain injury cases presented to this center over a period of one year, from 1st August 2023 to 31st July 2024.

All the traumatic brain injury patients aged over 16 years presenting to the hospital’s emergency and neurosurgery OPD were included in this study. The patients who were brought in dead, discharged on patient request (DOPR) and left against medical advice (LAMA); and treated through OPD, were not included in this study.

**Figure 1:**
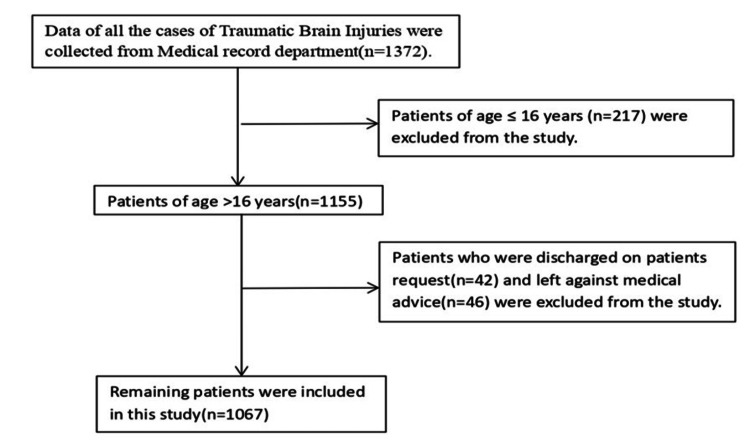
Flowchart showing inclusion and exclusion criteria for the study.

Figure 1 shows the total number of 1372 patients with traumatic brain injury admitted to this hospital’s neurosurgery department over one year, from 1st August 2023 to 31st July 2024 were studied and all the data were collected from the hospital’s medical record department. Patients below 17 years of age (n=217), discharged on patient request (n=42), and left against medical advice (n=46) were excluded from the study. Hence, a total of 1067 patients were included in this study.

Data collected include, but is not limited to, demographic details such as age, sex, address, and date of admission; details of injury such as mode of injury, GCS score on admission; radiological findings, management, hospital LOS i.e. days since admission to hospital till discharge, length of intensive care unit (ICU) stay, use of ventilator, operative procedures, GOS and mortality [9,10].

All the data were compiled on an Office Excel Sheet (v 2024; Microsoft, Redmond, WA, USA) and analyzed using SPSS Statistics version 30.0 (IBM Corp., Armonk, NY, USA). Categorical variables were reported as frequencies and percentages, while continuous variables were expressed as means and standard deviations. To assess the relationship between GCS at admission and patient outcomes, multinomial logistic regression was performed using GOS and LOS as dependent variables and GCS on admission as the predictor. Model fitting criteria and likelihood ratio tests were used to evaluate the significance of GCS on LOS and GOS. The statistical significance was set at p<0.05. 

As this was a retrospective analysis, no changes were made to the patient’s treatment. The decision to operate or manage conservatively was at the discretion of the consultant neurosurgeon caring for the patient. The study was approved by the Nepal Health Research Council (NHRC) under its expedited review process and an approval letter was provided with reference no. 49.

## Results

A total of 1067 patients were included in the study with the majority being males (n = 789, 73.9%). The most affected age group was 20 to 34 years, accounting for 38.4% (n = 410) of cases, followed by those aged 35 to 49 years (n = 295, 27.6%). Road traffic accidents (RTA) were the predominant cause of injury, responsible for 69.5% (n = 741) of presentation, with physical assaults and accidental injuries comprising 18.9% (n = 202) and 11.6% (n = 124) respectively (Table 1).

**Table 1:** Age and mode of injury; sex-wise distribution. RTA: road traffic accidents

Age and Mode of Injury	Male	Female	Total number	%
Age (in years)				
<20	91	8	99	9.3
20-34	335	75	410	38.4
35-49	185	110	295	27.6
50-64	128	66	194	18.2
>64	50	19	69	6.5
Total	789	278	1067	100
Mode of Injury				
RTA	547	194	741	69.5
Physical Assault	150	52	202	18.9
Accidental	92	32	124	11.6
Total	789	278	1067	100

At the time of admission, severity of brain injury was categorized based on patients' GCS scores. Most patients (n = 797, 74.7%) had mild traumatic brain injury (GCS 13-15). Moderate (GCS 9-12) and severe (GCS 3-8) injuries were seen in 14.1% (n = 151) and 11.2% (n = 119) of patients, respectively (Table 2).

**Table 2:** Glasgow Coma Scale (GCS) of patients on admission.

GCS on Admission	Number	%
3-8	119	11.2
9-12	151	14.1
13-15	797	74.7
Total	1067	100

Radiological assessment revealed that more than half of the patients (n = 552, 51.7%) had minor injuries (i.e. petechial haemorrhages and pneumocephalus) followed by subdural hematomas (SDH), being 14.2% (n = 152) on computed tomography (CT) head scan. Other less frequent injuries were epidural hematomas (EDH), subarachnoid haemorrhage (SAH)/intracerebral haemorrhage (ICH), diffuse axonal injury (DAI), and skull fracture, highlighting the variety of brain injuries seen in TBI patients. Neurosurgical intervention was required in 18.2% of cases (n = 194), with decompressive craniectomy being the most frequently performed procedure (n = 100, 51.5%), followed by craniotomy (n = 64, 33%). In most cases, undergoing decompressive craniectomy had SDH or EDH (Table 3).

**Table 3:** Types of management received by patients. GCS: Glasgow Coma Scale

Management	GCS on Admission				
	13-15	9-12	3-8	Total	%
Conservative	741	78	54	873	81.9
Neurosurgical Intervention	56	73	65	194	18.1
Total	797	151	119	1067	100

The average intensive care unit (ICU) stay increased with injury severity, with patients in the severe TBI group (GCS 3-8) more likely to require prolonged intensive care (more than seven days) along with ventilator support and undergoing tracheostomy. In contrast, patients with mild TBI (GCS 13-15) had significantly shorter ICU stay (less than seven days). The hospital stays showed a clear correlation with GCS on admission. Most patients with mild TBI (GCS 13-15) were discharged within a week (n = 686, 86%). Conversely, patients with severe TBI (GCS 3-8) tended to have more prolonged admission with more than two weeks (n = 55, 46.2%), and moderate TBI overlapping between them (Table 4). 

**Table 4:** Length of hospital stay based on Glasgow Coma Scale (GCS) on admission.

GCS on admission	Hospital length of stay (days)					
	0-3	4-7	8-14	15-30	>30	Total
13-15	350	336	91	20	0	797
9-12	6	20	48	64	13	151
3-8	21	20	23	38	17	119
Total	377	376	162	122	30	1067

Patients' outcomes were assessed using the GOS at discharge. Overall, 73.4% (n = 783) of patients had a good recovery, while 7.2% (76) died during their hospital stay. Among patients with GCS 13-15, 94.7% (n = 755) had a good or moderate recovery, with only 0.5% (n = 4) mortality. Conversely, in the severe TBI group (GCS 3-8), mortality was high (n = 61, 51.3%), and none achieved a good recovery (Table 5).

**Table 5:** Glasgow Outcome Scale based on Glasgow Coma Scale (GCS) on admission.

Glasgow Outcome Scale	GCS on Admission				
	13-15	9-12	3-8	Total	%
Death	4	11	61	76	7.2
Vegetative	0	23	39	62	5.8
Poor	6	39	13	58	5.4
Moderate	32	50	6	88	8.2
Good	755	28	0	783	73.4
Total	797	151	119	1067	100

**Figure 2:**
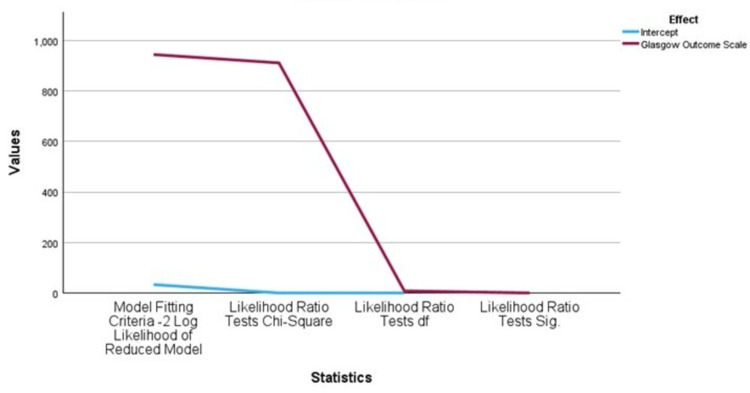
Likelihood test results for Glasgow Outcome Scale (GOS). Chi-square value = 910.851 with p < 0.001 makes it statistically significant.

**Figure 3:**
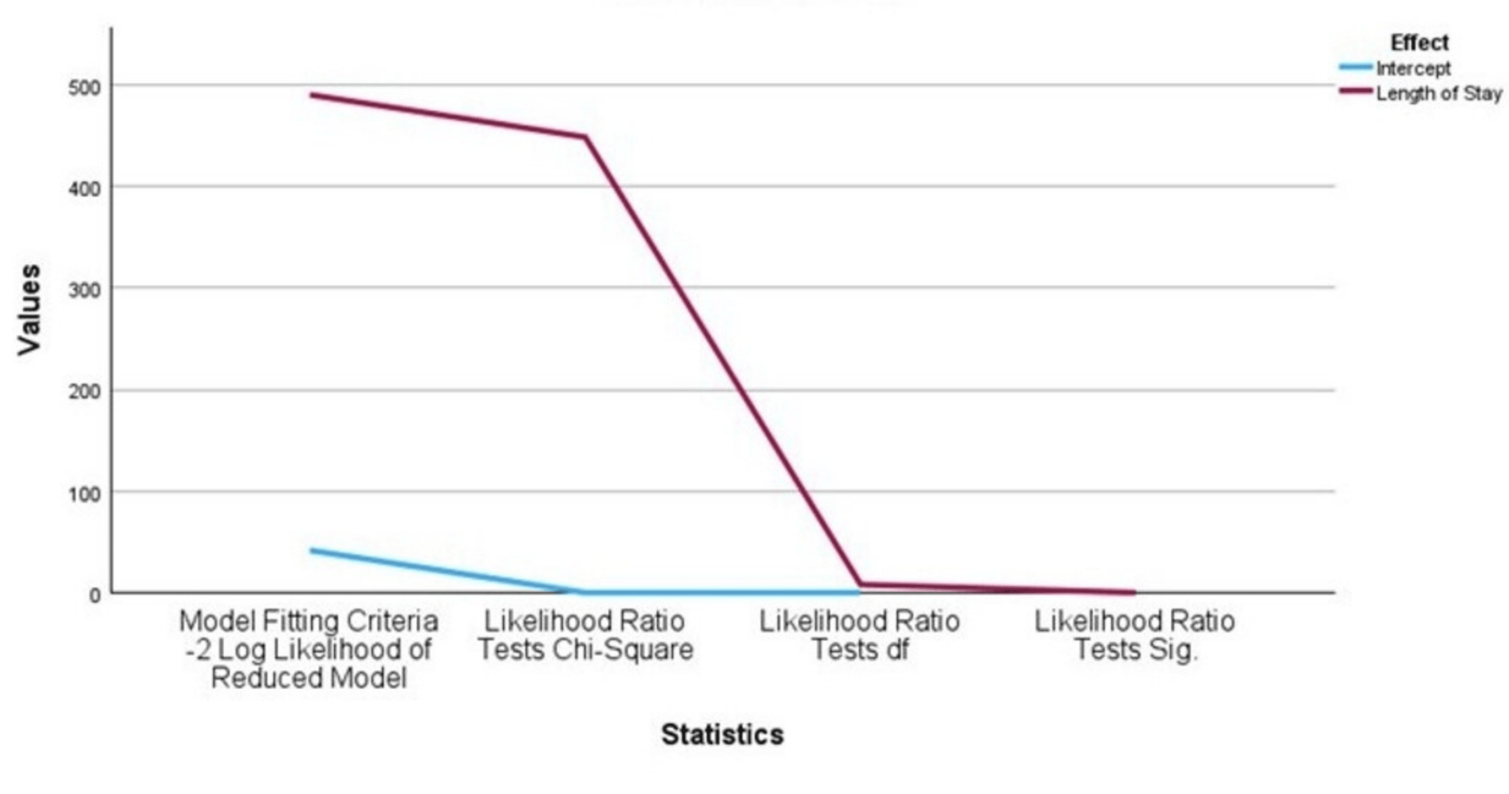
Likelihood test results for hospital length of stay (LOS). Chi-square value = 448.178 with p < 0.001 makes it statistically significant.

Statistical analysis confirmed a significant association between GCS on admission and both functional outcomes and hospital length of stay. In multinominal logistic regression, with severe TBI (GCS 3-8) as the reference group (OR = 1.0), the mild TBI (GCS 13-15) was associated with markedly reduced odds of death (OR = 1.13 x 10^-11^, 95% CI: 2.86 x 10^-12^ - 4.45 x 10^-11^, p<0.001) and poor disability (OR = 7.94 x 10^-11^, 95% CI: 2.08 x 10^-11^ - 3.03 x 10^-10^, p<0.001), while moderate TBI (GCS 9-12) also showed significantly lower odds of death (OR = 8.37 x 10^-10^, 95% CI: 2.89 x 10^-10^ - 2.42 x 10^-9^, p<0.001). The chi-square test for GOS yielded a highly significant result (χ2 = 910.85, p < 0.001), indicating that patients with lower GCS scores were substantially more likely to experience poorer outcomes or death (Figure 2). Regarding LOS, mild TBI predicted short stay (0-3 days: OR =2.42 x 10^9^, 95% CI: 1.20 10^9^ - 4.87 x 10^9^, p<0.001), whereas moderate TBI was associated with intermediate stays (8-14 days: OR = 2.73, 95% CI: 1.14-6.6.56, p = 0.025). The relationship between GCS and hospital length of stay was also significant (χ2 = 448.18, p < 0.001), with more severe injuries correlating with prolonged admissions (Figure 3).

## Discussion

This study provides a comprehensive analysis of the epidemiology, clinical presentation, and outcomes of TBIs at a tertiary care center in Nepal. It is reflective of global trends while offering localized insights into the distribution of TBIs. The majority of patients in our study were male, with the highest incidence among individuals aged 20-34 years. This mirrors global patterns, where the young adult male are often disproportionately affected due to factors such as road traffic accidents, which was the leading cause of TBI in our cohort, accounting for 69.5% of cases [11,12].

The predominance of mild TBIs, represented by a GCS score of 13-15 in 74.7% of cases, aligns with international data suggesting that mild injuries constitute the majority of TBIs treated globally [11-13]. The global incidence of TBIs is significantly driven by factors like road traffic crashes and falls, especially in low- and middle-income countries [11]. Our findings on RTAs being a major contributor are consistent with the data from the Global Burden of Disease Study 2016, which highlights the significant burden of TBI-related mortality and morbidity due to traffic accidents [1].

In terms of clinical outcomes, the correlations between GCS on admission and hospital length of stay as well as the GOS were statistically significant, reaffirming the predictive value of GCS in TBI prognosis [14,15]. These findings are supported by previous literature, which established GCS as a robust tool for predicting outcomes in severe head injuries [16-18]. Our study adds to this by demonstrating similar trends across all severity levels of TBI, where patients with lower GCS scores (3-8) had poorer outcomes and longer hospital stays; these findings should be interpreted as strong associations rather than definitive causal relationship.

The majority of cases in this cohort were managed conservatively. While this reflects the clinical spectrum of TBI presentations, it may also partly represent limitations in surgical and critical care resources in a low-resource setting, and this context should be considered when interpreting management patterns. The direct and indirect costs associated with TBIs are substantial, as they require prolonged hospitalization, neurosurgical interventions, and, in severe cases, intensive care unit stay and ventilator support, as seen in our study. The economic impact of TBIs is a global issue. TBIs contribute to significant long-term healthcare costs, including rehabilitation, loss of productivity, and the need for long-term care in case of severe injury [6]. This not only burdens healthcare resources but also places a financial strain on the families of patients, many of whom may already face economic hardships. The World Health Organization estimates that RTAs cost countries approximately 3% of their gross domestic product (GDP) annually [19,20].

The mortality rate in our study was 7.2%, which is slightly lower than some other studies conducted in similar settings. This could be attributed to improvements in emergency response and neurosurgical interventions, such as decompressive craniectomy, which was the most common surgical procedure performed. Previous studies have shown that early surgical intervention significantly reduces mortality in cases of extradural hematoma, supporting our findings of the importance of timely neurosurgical management [15].

While this study offers valuable insights, there are limitations. As a single-center retrospective study, the results may not be generalized to other settings, particularly those with different trauma management protocols. Additionally, patients who left against medical advice discharged on patient request, or treated as outpatients were excluded, which may introduce selection bias. Finally, time from injury to hospital presentation was unavailable, which may have influenced outcomes. Future studies should focus on multicenter data and incorporate long-term outcomes to provide a more comprehensive understanding of TBI epidemiology and management in low-resource settings.

## Conclusions

This retrospective study has provided a detailed examination of the epidemiology and outcomes of traumatic brain injury at a tertiary care center in Nepal, emphasizing the significant burden that TBIs place on both healthcare systems and the economy. Road traffic accidents were identified as the leading cause of TBI, and the GCS on admission was found to be a strong predictor of patient outcomes, including hospital length of stay and clinical recovery. However, the financial strain associated with prolonged hospitalization, intensive care, and long-term rehabilitation cannot be ignored, particularly in countries where healthcare resources are limited.

Given that the majority of cases were managed conservatively, there is an opportunity to invest in preventive measures like enhancing road safety, implementing more robust traffic regulations, and improving emergency trauma care are essential to reducing the incidence of TBIs. Furthermore, standardized outcome prediction tools such as GCS should be widely adopted to improve patient management and optimize the use of healthcare resources. Future research should focus on multicenter studies that include long-term follow-up to better understand the broader implications of TBIs on public health and the economy, especially in resource-limited settings like Nepal.
